# Intemperance Considered as a Form of Mental Disorder

**Published:** 1858-01-01

**Authors:** 


### Art. VI.—INTEMPERANCE CONSIDERED AS A FORM OF MENTAL DISORDER.

There cannot be a more fertile subject for the student of psychology than to elucidate the direct and indirect consequences of intemperance, not only for its ravages on the vital organs generally, but for its special injurious effects on the mental faculties, and as a predisposing cause of many forms of insanity.

We shall examine some of the facts to prove that excessive intemperance tends, not only to injure those who indulge in this habit, but that it also affects their offspring, occasioning an excessive craving in them for the gratification of similar tastes, which is often so irresistible as to constitute a special form of disease.

It is, therefore, essential to trace the *modus operandi* of alcoholic poison on the system, which induces, besides the above consequences, many forms of moral depravity.

This latter view is obvious by an inspection of our jails and workhouses, which present ample proof of its devastating effects on the higher attributes of man; and our infirmaries and hospitals will furnish indubitable evidence that it predisposes to many forms of corporeal disease; whilst our county lunatic asylums present many lamentable cases of mental affections induced by excessive intemperance.

Lastly, it is now a well-established fact, that all those abnormal conditions are transmitted as heirlooms by inebriates to their unfortunate children.

In one short paper we can give little more than a mere outline of the subject, but yet sufficient to show that the evils which will be enumerated are not exaggerated for any special purpose. They are patent to every observer, and demand not only the sympathy of philanthropic men, but some effort to prevent their continuation. Finally, we shall submit a few reflections, in order to show how this result may be accomplished by means similar to those made use of to cure various mental affections.

So that for our purpose there is not needed any novel treatment or startling new views. The inferences will be deduced from the premises, and may therefore be regarded as simply consistent; for, prior to submitting our deductions, we shall endeavour to prove that *drunkenness* in some of its forms should be treated as a type of insanity, and that in all inveterate cases the victims should be regarded as patients to be placed under restraint, and forcibly prevented from continuing their debased habits!

It will be acknowledged that intemperance is a leveller! All its votaries, whether illiterate or learned, rich or poor, are brought down to the same low moral condition. And just for this reason, it acts on the mind through the organization in general, and the brain in particular. And if we select an example of one highly cultivated, we have the advantage of his experience of its mind-destroying tendency. Its continued excess and the fatal consequences are thus graphically described:—

. . . . "'till the brain became

In its own eddy boiling, and o'erwrought,

*A whirling gulph of phantasy and flame.*"

The effects on a worshipper of this modern Moloch is unmistakable havoc. He is rendered abject and reckless, and qualified for deeds of violence; daily he is rendered more savage and infuriated, and becomes desperate and dangerous even to his habitual associates. His criminal outrages are perpetrated under a certain amount of excitement, but he abstains from great excess until he has performed his immoral ravages, and then "he drinks deep," and "steeps his senses in forgetfulness." He is, under such circumstances, impotent to act; his expression is then most idiotic, his appearance desolate, and if he attempts to move, he is in danger of clasping his kindred clod, and presents a being of such disfigured form, as if he had lost every trait of humanity. And in verity it is difficult to believe one so uneducated and so brutalized had ever felt the pure and refined emotions arising from moral perceptions; or that he had ever experienced the love of the beautiful and the true, by contemplating the works of creation. We shudder at beholding the mental degradation of an immortal agent, and we come to the conclusion that Government, in its paternal character, is bound to use means to prevent the great mass of the working population becoming similar to the sketch we have made of one slave to intemperance, with its necessary concomitant, crime! Intemperance is indeed a giant vice, and requires commensurate means to prevent its spreading,—means only possessed by the rulers of the country; and if they neglect their duty, let them not imagine that even the most innocent, pure, and holy will not suffer from the malignant consequences thus unrestrained; and rendered liable, whatever their station, to be injured in the midst of such a vortex of ruin.

It so corrupts, that servants sacrifice their integrity to it, and violate the misplaced confidence of their employers; and it pollutes the minds of women, that nurse-maids may corrupt the innocent beings under their charge.

So that even as individuals we dare not remain unconcerned spectators; we must combine to stop the torrent of evils which are more devastating than pestilence and plague. We pity the obtuseness of those who consider that, although it may be the source of some crimes, the revenue could not dispense with the sale of that which induces intemperance. Verily they have eyes and see not, ears, and hear not; for the injuries to life and property are more than commensurate to the monetary advantages, and that, if it were possible to prevent altogether the sale of alcoholic compounds, there would be a vast amount of property saved, besides the absolute millions sacrificed for their purchase. And in these advantages we have not added that there would be prevented a number of bad husbands, fathers, mothers, wives, sons, and daughters, which are made by intemperance, and if a list of them could be annually exposed to public gaze it might startle and probably deter some few of the noviciates of this fearful habit. Adding to these brief facts the searing effects of intemperance, and that its tendency is "to drain every drop of the milk of human kindness" from the hearts of its votaries, and then leave them steeped and saturated with extreme and inveterate selfishness, which is confirmed at every session for the trial of criminals.

These are simple truisms, for inebriating stimulants damage the organization, render the moral sentiments callous, and prostrates the God-like attribute of reason, often driving its worshippers to madness or murder!

There requires little reasoning to explain why such must be the ultimate results, as the primary effect of alcohol is to increase the circulation of the blood; in the first stage brightening the eyes, and exciting symptoms of greater vivacity, as the mental faculties become stimulated. If the doses are continued, there is soon manifested a vast change in the expression—the eyes become slightly injected, the face flushed, the mouth hot and dry, the body feverish, and the brain congested. This latter state is indicated by a sense of heaviness and stupor. If, despite these warnings, additional draughts are quaffed, the individual is in that ominous condition approximating to actual disease.

We shall, after these general statements, submit some few particulars under the following heads:—

1. The physical and moral ravages of intemperance.
2. The intellectual lesions.
3. That the inordinate craving for alcohol is an hereditary affection.
4. That drunkenness must be regarded as itself a form of insanity, irrespective of delirium tremens.
5. Concluding reflections.

It is well known that the stomach of the drunkard suffers from the effect of his excesses, and that its functions are much impaired, so that it can ill perform its allotted task.

The proof of all these effects is graphically expressed in the countenance; for the inebriate either looks very pale and pasty, or else bloated with an accumulation of diseased fat. In the latter case, the slightest scratch, which would be unheeded by a healthy man, will often induce mortification and death.

But if the drunkard does not become unwieldly, then he has a cadaverous expression, with, at times, feverish and hectic symptoms. The intemperate are also liable to affections of the liver, bladder, kidneys, and so forth; and he pays the penalty of his excesses by all the complications of dyspepsia, and by having to endure the racking, gnawing pains of gout.

Besides these diseased conditions, the thoracic organs are implicated. The heart or its valves may become ossified, or it may lose its contractile power in some degree, producing many distressing consequences ; and if the inebriate has inherited weak lungs, his career may be cut short by rapidly developed consumption.* It is patent to every practitioner that the tendencies of these diseased organs may be transmitted to the children of the intemperate besides the other affections to which we shall subsequently allude.

These brief statements will suffice our present purpose, as we merely wished to indicate that intemperance, among its other evils, deranges the whole of the chylypoietic viscera, involving the organic functions. It also disfigures the outward form, but this latter is of less consequence than the changes induced by this vice on the inner life of the individual.

Before we treat on this important part of our subject, we must call attention to the fact, that the fatal habit of intemperance injures the nervous system generally, and the brain in particular.

A vast many of the patients attending the ophthalmic institutions are either drunkards or their children, and the disorders they suffer are, inflammation of the eyes, amaurosis, and very often loss of sight. Many also suffer from deafness and the absolute loss of the senses of *taste* and *smell.*

Writers on the deaf mutes attribute the affection to intemperance as one of the causes. And it also induces paralysis, epilepsy, and apoplexy.

Whilst the brain gives surety of its functional disturbance in delirium tremens, which specially is induced by intemperance, in this affection the victim sees the most frightful objects mocking him, or threatening him with ribald jests or horrid denunciations, and often exciting in him a sense of terror from their demoniac expressions.

We will only cite one case as an instance that, after the active attack, the hallucination still, at times, annoyed and irritated the individual.

Mr. R—— was a drinker of brandy to great excess, and although a man of great talent, he had lost all moral control over himself. During one of his attacks of delirium tremens, instead of threatening creditors stunning him with their demands, he fancied that a large black raven was pecking at his right shoulder.

* Drunkards who have scrofula transmit this dreadful disease in an aggravated form ; whilst they are also liable to jaundice and fits of melancholy depression, which, when inherited, is a predisposing cause of suicide.

This made him rave and swear most frightfully. He, however, recovered from the attack, and resumed his daily potations; and when he had imbibed a certain dose, the old black raven would again annoy him. As he was a public man we often saw him, and frequently noticed that in the midst of an intellectual discourse he would turn his head abruptly towards the right shoulder, and say in a half smothered oath, "Be still; be quiet, will you?"

So, one day we asked his man, why Mr. R—— did so? "Why sir, don't you know that he still thinks his old enemy, the black raven, is pecking at his shoulder; but he is never troubled with this fancy until he is nearly drunk; and," he continued, "it takes a rare quantity of brandy before he is so."

He died in the prime of life, suffering in the most fearful manner, bodily and mentally.

But before this lamentable malady takes place, the inebriate is forewarned by some abnormal state of some of the external senses.

These latter statements we could verify by many instructive cases; we select the following:—

Mr. M—— was a most inveterate drinker from his youth, but was mild and gentlemanly when sober, and full of regret at the madness of his career; and yet he continued this suicidal habit.

About a year after we had seen him he complained of an absolute loss of his *smell* and *taste*, and was strongly urged to abstain from all intoxicating beverages. He made a powerful effort to do so, and partially recovered his lost senses. But real or fancied annoyance made him relapse, and then he was unable to distinguish the most fragrant from the most fœtid substances. And yet, so enslaved had he become to his fatal habit, that he continued to indulge in excess, to use his own apology, "to prevent the irritation he experienced, by stupifying his thoughts." And this he continued to do until active disease of the brain took place, and after repeated attacks he died of delirium tremens.

The next case we will submit, from its "pointing a moral," if it will not "adorn a tale," might have terminated similarly, if the poor inebriate had not been cut off in his career rather prematurely by a most unfortunate accident.

Mr. B—— had been in business as a respectable retail druggist, at ——, and was said to have been intemperate as a young man, but for some years after his marriage he restrained the strong propensity; yet from some circumstance we are unacquainted with he began his old course, and soon became an inveterate drunkard. All his stock and furniture were disposed of to gratify his inordinate craving, and his wife and children were reduced to poverty.

The ravages he committed on himself were written in red and blue blotches on his face and nose, but these were trifling to his absolute loss of both *smell* and *taste;* still he might be seen reeling about the streets whenever he could procure money for drink.

When most degraded in mind and body he was urged, in a moment of sobriety, to take the temperance pledge, and he soon recovered some more natural expression, and a little feeling of renewed respectability; but he was still deprived of *smell* and *taste.*

A situation was procured for him at the house of a respectable firm of wholesale druggists, in what we believe is called the DRY department. Yet, with all his past experience, there still lurked a craving for a more potent stimulus than coffee or tea, and so he was easily persuaded to break his pledge.

All kinds of alcoholic beverages were excluded by the firm from their establishment. Yet this salutary order was evaded, and spirits or porter were procured in medicine bottles. This evasion cost the life of an individual. Poor B—— had clubbed for some porter, and during the time it was sent for a bottle of laudanum had been placed on his counter, to be enclosed in a parcel he had to pack. This bottle he mistook for the forbidden porter, and took a hearty draught of it, and immediately recognised by his sensations the fatal mistake. A stomach-pump was instantly procured, and every effort which science or humanity could apply to save him was tried, but all proved useless, and, if our memory is correct, he died within half an hour!

As he had not recovered his *smell* or *taste*, and had recommenced his former intemperance, there is little doubt but that he would have ultimately been carried off by some form of cerebral disease.*

We will now briefly advert to some effects of intemperance on the moral perceptions.

It is proverbial "that fools and drunkards betray their natural tendencies;" for they cannot conceal their follies or their vices.

If the inebriate is naturally of an irritable temper, he is sure to quarrel under the influence of drink. If he is sly and cunning, he will become suspicious and spiteful, and often very mischievous. Pot-companions have frequent brawls; and the friendship of drinking associates is held on a very slight tenure. Look at the

* These two cases will assume an importance in our elucidation of the organs implicated in the hereditary intemperance, and if our views are rejected it would be impossible to explain how a craving for intoxicating drinks can exist when the drinker had neither *smell* nor *taste!*

violated confidence; the adultery; manslaughter; the breaches of commercial faith, even amongst the comparatively educated;* also the wife-beating and other brutalities among the working-classes; and thefts, burglaries, murders, rape, and so forth, among the degraded portion of the community—those who are without any culture of their mental faculties. And thus we possess a mass of evidence, amounting to actual proof, that intemperance merely rouses into frightful activity the lowest of human propensities.

This is confirmed by the fact, that it is rarely the case that drunkards are chaste and highly moral in their conversation and actions.

It is therefore a natural consequence that intemperance should more or less affect the brain, and induce many forms of insanity. This is a valid reason to avoid the temptation and the penalty; yet it is a lamentable fact, that those who become addicted to this debasing habit cannot be deterred from it by the most urgent appeals to their better nature, and the most startling narratives of the diseases it induces. To such infatuated beings, it is most true that—

> "Every charm of gentler eloquences
> All perishable—like the electric fire—
> But strike the frame, and as it strikes, expire!"

We shall, at the conclusion of this essay, submit some reasons for the use of other means than either precepts or arguments, to stem the frightfully increasing evil of intemperance.

We have, therefore, the most voluminous evidence that apoplexy and delirium tremens are not the only affections consequent on the baneful vice of inebriation; that it is not only destructive to the physical and moral health, but that it also prevents intellectual development.

The following case corroborates this statement:—In the town of B——, in Suffolk, there resided a married pair, who lived on a small independence. They were near relations, and were reported to be not very remarkable for great intellectuality; and from their mode of life it would seem they did not use what little they had. They rather preferred gratifying their animal propensities, and miserable were the consequences. "Their usual occupation," says our informant, "consisted in muddling their brains with vinous potations, probably never getting absolutely intoxicated, but in that state usually designated 'fuddled.' In this condition they retired every night, and the results of their depraved habits soon became manifest. They had five children,

* We do not regard any one educated if the moral attributes are left untrained, and therefore not manifesting any influence on the character and conduct.

all of whom were of the worst class of idiots; that is, not only defective in intellectual capacity, but they also inherited from their progenitors the animal propensities with an intensity of power."

Similar examples are patent to every physician, for intemperance produces its havoc as a mind-destroyer in all places. Look, for instance, at the squalid groups lounging at the "gin-palaces" in this metropolis and in all large towns; and mothers may be seen (O profanation to this sacred name!) standing or reeling with their weak, sickly, and emaciated children, many of them, from want and neglect, staring in vacant idiocy.

But the most startling problem connected with intemperance is, that not only does it affect the health, morals, and intelligence of the offspring of its votaries, *but they also inherit the fatal tendency and feel a craving for the very beverages which have acted as poisons on their system from the commencement of their being!*

The first time this aspect of the subject was forced on our attention we had become acquainted with persons in respectable positions of society who would break out for a week or so, and during the time would continue to drink to great excess, after which they would remain for some lengthened period rigidly sober.

We at first attributed the latter phase as the consequence of the nausea and derangement of the system, but we soon had a mass of evidence which, in a great measure, rendered this inference invalid; for we noticed that the alternate inebriety and abstinence assumed a regular periodicity.

We also met with cases which confirmed our first impression, as to the tendency being transmitted "from sire to son." Two remarkable instances were related in a public assembly. Two respectable men, members of two different denominations of religion, had made the discovery in their youth that they experienced an irresistible craving for alcoholic liquors, but that they could not taste them without continuing to drink until they became inebriated. They were men of good sense, and very moral, and they determined to act on their higher motives, and abstain altogether from the use of these fatal beverages. This they did many years before the evils of intemperance had induced men to form societies for its suppression.

These two rational reformers continued water drinkers for many years; but both were induced, on some special occasion, to taste "the forbidden beverages," and each is reported to have had some misgiving as to the danger and hazard of the experiment, but they were overruled, and yielded to the fatal temptation. For it seems the moment they drank all their long-smothered latent craving was revived with fearful intensity, and both, on the first occasion, became intoxicated; and then they continued their old course, and both ultimately died in mad-houses, no doubt expedited by the remembrance of their long-sustained victory over this vicious tendency, and the weakness and degradation of ultimately being vanquished by it.

From similar well-authenticated facts, we were induced to believe that drunkenness, like many other morbid affections, is hereditary; and then we naturally sought for a solution—How is this tendency transmitted?

Before submitting some of the evidence in our possession, by which both questions may be answered, we may premise that all the animal functions are under the directing influence of the brain; that, for instance, the cerebellum conserves the sexual functions, and that all the feelings and sentiments, included under what are termed "the affective faculties," are located in the cerebrum. We merely insist on this induction to strengthen the *data* for the following elucidation.

That in order, for example, to comprehend how the tendency of intemperance exists, we must avail ourselves of the assumed fact, that a portion of the brain gives man "a desire for food and drink," so that these essentials for his health may not be left to accident. Spurgheim called this organ "Alimentiveness;" whilst other writers term it "Gustativeness," or "Instinct of hunger and thirst." And it will be by the physiological evidence of this function that we are enabled to explain and answer both queries.

Nay, we will affirm that, if we reject the evidence for the existence of such an instinct, though the facts of this craving for intoxicating drinks be admitted, it could never be explained. We might say that it was very curious, or very mysterious, and endeavour to invent some ingenious speculation to account for it; yet it would be, under such treatment, a tangled web of mere gratuitous assumption, or unsupported ambiguities.* The desire for food and drink are appetites possessed by all animals; and there is a periodical demand for both, to keep the body in a state of working order. Observations, often repeated, enable us to point out the locality of the organ of *alimentiveness*, it being

* This was just the dilemma in which the late highly-gifted Sir Charles Bell was placed in his "Bridgewater Treatise on the Hand." He could not reconcile the great mechanical tact of man to have been bestowed on him merely by the hand, and so he had to admit "that there must be some portion of the brain destined to give him his manual dexterity;" and this portion of the cerebrum he called "*The Muscular Sense!*" all of which ingenuity he would have been spared had he admitted "The Organ of Constructiveness," which is said to be more or less developed in the ratio of the manipulative power.

situated at the base of the brain, and occupies the temporal fossa on each side of the head. It gives origin to the olfactory nerves. When the organ is large, there is great width at the lower part of the skull, just at the temples, and above the zygomatic arches. This may be verified by the inspection of all voracious animals, such as the lion, tiger, pig, and so forth.

The same parts of the head are generally very full in gluttons and drunkards, but they are narrow in persons who have perfect control over their appetites.

As presumptive evidence of such an instinct, it may be affirmed that the stomach does not select food or indicate any preference, and, although it is often a very ill-used organ, and greatly imposed on, it has but one remedy—it can reject the superabundant supply.

In a state of nature man and animals would, as a general rule, select the sort of food most suited for them, by the sense of smell, but the stomach is, and would be, a mere passive recipient.

But still it might be said, supposing these statements to be correct, they furnish no positive evidence that the selection of nutriment is the province and prerogative of *alimentiveness.*

We might content ourselves with simply answering that the two cases of the absolute loss of the senses of *smell* and *taste,* and the continued craving for what could not gratify by the flavour or the odour, must have been excited by the remembered instinct which had set up some preferences, not from tradition, but from the primary gratification these drinks imparted in the commencement of their career.

We, however, submit another kind of evidence, and which gives a complete answer by fair induction.

1. That *alimentiveness* indicates a regular periodicity.

2. This is proved by the fact that whatever may occupy the mind of an individual, he is apprised of his meal-time, by an urgent desire for food.

3. That this periodical craving cannot originate in the stomach itself, for should any casualty prevent food to be eaten, there is little or no inclination to eat.

4. If, therefore, the periodical instinctive desire for food depended on the stomach, as it remains empty, the desire would be indefinitely persisted in. But as this is not the case, we are forced to conclude that *alimentiveness* is the "Master of the Ceremonies."

5. It should also be remembered that the desire to eat may be induced by savoury smells, which, affecting the olfactories, the excitement is transmitted to the organ of alimentiveness, and thus the craving is so great, that a keen appetite is experienced.

Lastly. We are, from these premises, warranted to conclude that the stomach does not induce the desire (as inferred from the fact mentioned); and further, when the next meal-time arrives, there will be experienced again a great desire for food. We might add other well-ascertained facts—that, for instance, insane patients who try to starve themselves have moderate *alimentiveness;* whilst criminals left for execution will, when this organ is large, be very eager for their last breakfast, and enjoy it with as much *gusto* as if nothing unpleasant was anticipated.

The inordinate exercise of any feeling or instinct induces not only greater activity, but also greater intensity of power.*

We therefore shall submit evidence that a craving for inebriating liquors becomes, from this circumstance, an hereditary affection; just as in the same manner when individuals exercise certain of the perceptive powers the tendency is transmitted, and hence there are families of mechanicians, painters, sculptors, &c.

We have already mentioned—and only repeat it now as affording collateral proof—that the base of the brain is the seat of the animal propensities, and that our passions are stimulated by strong drinks; for they excite the heart's action, and the blood is propelled with an accelerated force to the brain generally, and to the base of the brain in particular, where are situated the largest blood-vessels—so there is not any marvel that the feelings are roused, whilst the more remotely situated powers are scarcely affected. And hence the habitually intemperate do not manifest any high and exalted sentiments; recklessness takes the place of prudence, and every pure affection is trampled on and outraged. And although the inebriate is at times conscious of the destructive and searing influence of his debased habit, he seems like one spell-bound, and if he has not inherited this horrid insanity, he is sure to transmit it to his family.

Mr. J—— was a professor of religion, but the truths he inculcated were rendered useless by his own intemperate habits. His children received them with mockery from their heavy-tongued and scarcely articulate father. Morality taught by him acted as a few drops of water on a dry and barren ground, and excited the ridicule and not the reverence of his hearers. But he never was seen reeling drunk—only constantly kept up to the point of saturation. He was cut off in the prime of life.

* We have numerous instructive facts in reference to the instinct of *alimentiveness* in cases of gluttony, both in persons reputed intelligent, and those who were idiotic or insane, and many curious *idiosyncrasies.* These we may on some future occasion be disposed to prepare for this Journal; there are many psychological phenomena connected with the subject.

His wife had a similar tendency; she had a very bad "stomach complaint," and she took spirit as a balm; and she imbibed it so continually, that scandal affirmed "she liked the medicine."

Was it therefore any wonder that all the younger J——s became drunkards?

We would not use this case as furnishing proof of the hereditary tendency of drunkenness, as it might be referred by some to the children having been educated to drink. But we knew some of the family, who were witty, intelligent, and most excellent companions, being chided for their suicidal conduct, say, "We can't help it; we inherit a strong love for rum or gin!" One actually bound himself by some heavy penalty, and after some months' abstinence, broke out and declared "The craving was actual torture—he could not help himself."

The mother died a confirmed drunkard, and so did every one of her children.

Mr. B——, of ——, Yorkshire, had also a large family, but he and his wife were never exactly sober. Soon after we knew them, the lady died suddenly, as it was reported, of a fever, but in fact from an attack of delirium tremens; and a few hours before the affection came on, an empty brandy bottle was found under her pillow.

The old man had an iron constitution, and stood for some years the havoc of this searing vice; but he lived long enough to witness the sad effects on his family. His eldest son, under the influence of alcohol, committed suicide, and all the other children (with one exception) came to an untimely end, all being inveterate drunkards; but the final blow to the poor old sinner was struck by his only daughter, as she was brought home by the police in a state of inebriety. This shock was too much, and he did not survive it.

We could narrate a vast many cases, some of literary and professional men, who, in answer to our query whether they had a strong craving for intemperance in drink (judging so from their large alimentiveness), have either confessed themselves the slaves of this propensity, or, to avoid the baneful consequences, have shrunk from the temptation; "for," said one, "if I drink at all, I cannot help going on to maddening excess—*and my father was so before me!*"

In a Journal devoted to investigate the phases of insanity, we need not insist on the fact that literally "the sins of the fathers (physical and moral) are visited on the children;" and we also affirm that the drunkard's propensity is also transmitted to them through some morbid condition of the alimentive organ.

We possess a large mass of evidence in proof of this statement, as valid as that the brain, heart, lungs, stomach, bladder, and kidneys are implicated under long-continued acts of intemperance.

When once this vice assumes a mastery, the slavery to it is most humiliating. There is only one chance of curing this form of disease in its incipient stage—to warn the victim that he stands on a precipice which is full of danger, and that, if he has not destroyed his intellect, he will make an effort to save himself from the imminent consequences. That if he does not make this effort from a high moral motive, he should pause ere he drags to certain destruction the innocent and helpless beings whom he has been instrumental to usher into existence, and which it is his sacred duty to cherish and train, that they may be fitted to perform their parts on the busy stage of life.

He might be told that, instead of being selfishly reckless, he should rigidly observe the laws of health, and studiously exhibit for their example the most correct moral discipline, and should cultivate his own intellect, that he might be more fitted to train and educate his precious charges.

And, as a practical lesson, he should have his attention called to the fact, that in mercy many premonitory warnings were given him when he violated the laws of temperance; that his head ached and temples throbbed; that he had a fevered skin, and hot, dry mouth; that the nausea and acidity he experienced, as ordinary indications of physical disturbance, should be heeded, and that he should also note his mental disturbances; that he was irritable at trifles, and quarrelsome without any cause; then, if he continued his career, and seemed incapable of perceiving so many body-and-mind-destroying influences, he should be then treated as a madman, and be forcibly prevented from injuring himself or those dependent on him.

We are disposed to consider, with Dr. Caldwell,* *that drunkenness is, in some of its types, itself a species of insanity!* Its tendency bears a marked analogy to the diagnotic symptoms of other forms of this disease. We speak not merely of its chronic forms; but under the potent influence of ardent spirits it may be suddenly developed, and as suddenly cured. We cite the following instructive cases:—

During our residence in Yorkshire, we paid a visit to ——, a small place in the West Riding, and was told by a very intelligent person of a small farmer of the name of P——, a married man; that he seemed to love his wife and child very

* "Thoughts on the Pathology, Prevention, and Treatment of Intemperance, as a form of Mental Derangement." By Charles Caldwell, M.D., Professor of the Institutes of Medicine and Chemical Practice in Transylvania University.

much; and yet, under the influence of drink, he had suddenly made a recent attempt to destroy them both. It appeared that the farmer had been very temperate; but he had, on the same day of which the fearful occurrence took place in the evening, been drinking many glasses of spirits and water with some one whom he had not seen for years, and that he returned home very drunk, and in a state of great irritability of temper. His very amiable wife did not even rebuke him for his disgraceful excess, yet he did nothing but quarrel and abuse her; and when at last her womanly nature protested against his words of unjust reproof, he rose like a maniac, and struck her so violently that she fell on the floor, and for a few seconds lost her consciousness. He was furious, and struck himself, vowing vengeance against every one, confirming the words of the poet—

"That to be wroth with those we love,
Doth work like madness on the brain."

The woman was still prostrate, and contemplating the strange metamorphosis of him who had hitherto been a most affectionate partner, when she was astounded by another phase—he, with a frenzied expression, charged her with a criminal intercourse with some ideal personage, and then he seized the cradle in which their son was sleeping, and placed it on the fire. She gave a most piercing shriek, but still, nerved with strength and presence of mind, she took the infant from the grate, and holding the blackened cradle with its contents before her mad husband, she said, with a wild and husky voice, "What is the matter with you? You tried not only to kill me, but you have attempted to murder our poor little innocent Billy! May God forgive you!"

The neighbours had witnessed this painful scene, and they took, in silent admiration, the cradle from this heroic mother; but were shocked and surprised at the conduct of her husband. The violence of her grief, and the suddenness of the whole horrid affair, had sobered him, and he sat with his hands before his face, crying and sobbing, "May God forgive me!"

A reconciliation took place, and he expressed the deepest contrition; and in the full and sincere repentance of his heart, explained that the drink he had taken quite maddened him, and had nearly led him to the perpetration of a double murder.

In this case we have an instance of sudden insanity from the potent poison; and, under a new excitement, an equally rapid recovery.

We select another from a police report, which occurred a very few years since in this metropolis:—

"A woman, in an attic in some low locality, attempted to throw her son, a boy about five years old, out of the window in her room; and his screams brought a neighbour, who lived in the adjoining apartment, just in time to save the child. The unnatural mother, who was in a state of inebriation, was given into the custody of a policeman, by whom she was taken to the station-house, and locked in a cell all night. The next morning the case was heard before the sitting magistrate, when the principal witness said (after telling the above incident), that she had not a doubt that the wretched woman had been induced to kill her son, that she might pledge his shoes for a little more gin! And she added, that when she was sober, she was rather an indulgent parent; but that when she was drunk she was like a mad woman, or words to this effect.

"The magistrate then asked the trembling and contrite prisoner if what had been related was the truth? and if she had any questions to put to the witness? She shook her head, and acknowledged that she had no other thought than to get some more liquor with the shoes, and that she was always demented when she had drink! If she had not been so, she was sure she would not hurt a hair of her dear boy's head!"

The worthy magistrate gave her a most excellent lesson for her future conduct, and she was discharged on her own sureties not to repeat the offence.*

We could give many similar instances where "a mother's love," the strongest instinct of woman's nature, has been withered by intemperate habits; and that such mothers, instead of making sacrifices for the preservation of their offspring (sacrifices which such beings will always make in a normal state of mind), will act in violation of every sentiment of humanity to gratify their craving for an irritating poison which maddens them.

Can any other form of disease but insanity express this sad and degraded condition? And these instances suggest the best curative process. In the one instance (Farmer P——), he had been a sober man, and the sudden excess induced the temporary affection; but as he afterwards rigidly avoided the exciting cause, he continued to retain his sanity. In the other instance: though under the maddening stimulus the mother attempted to murder her son, yet a few hours' abstinence had so changed her that she felt her degradation, and the enormity of her contemplated crime.

We therefore cordially agree with Dr. Caldwell, that all inveterate and dangerous drunkards should be treated as insane; then they would be saved from committing crime, and be forced to abstain from that which maddens them. And although there might be some persons who would thus be under the necessity of remaining in seclusion, yet others might, by a curative process, be ultimately restored to society, and avoid any relapse by abstaining from its predisposing cause.†

* We report this from memory, not having the paper in our possession.

† Dr. Caldwell endeavours to show what kind of affection is drunkenness—what

This would be a wise and judicious proceeding, for an inveterate criminal intemperance will often so degrade its victims as to render them useless to any one—if among the higher classes, embittering the feelings of relatives; and in the more degraded portion of the community, rendering them a tax on the more industrious.

The argument is strengthened by the fact, that frequently the habitual drunkards will, even when the intellect is weakened and the limbs paralyzed, still persist in their destructive career! Shall this outrage against humanity be continued? Would it not be conservative to such degraded persons to forcibly save them from indulging in their senseless and suicidal habit? Would it not be an act of mercy to place them in some institution, where time and medical treatment might restore them to a more normal state?

The wealthy who are reduced to such a condition may be removed from the public gaze, and may thus avoid the scoffs of an idle and unfeeling crowd, because their friends have the means of placing them in some private lunatic asylum; but the poor worn-out debauchee, whose lot is extreme poverty, whose home (if such a miserable place can be so called) is destitute of means—he must linger out his cheerless existence, naked and shivering, or accept the last refuge for the destitute—the parish workhouse!

The penalty which is inflicted on the rich and the poor for their intemperate habits is marked by special differences, if we examine the different results on their respective families.

The children of the rich inebriate may be weak and nervous, yet those who have to superintend their education may modify and improve their bodily and mental condition. They have the advantage of an ample supply of food, and care is taken to improve their intellectual and moral perceptions by systematic mental culture.

But the offspring of drunken parents, sunk low into the very depths of poverty, have the horror of their condition aggravated effect it has on human health—wherein consists its ungovernable appetite for ardent spirits, and how the entire evil may be prevented or removed. "Drunkenness," he considers, "consists of an affection of the brain—the spinal nerves being also implicated—but chiefly as an affection of the part of the brain belonging to the animal propensities, and hence its first effect in rousing passions and animal desires." The appetite for intoxicating liquors is regarded by Dr. Caldwell as springing from a morbid excitement of the organ of *alimentiveness;* and he explains the augmented intensity which attends indulgence, by reference to the ordinary principle of exercise invigorating the power." Dr. Caldwell, therefore, recommends "bleeding, tartar-emetic, cold applications to the head, purging, and spare living. By these means the paroxysm is shortened, and by their repetition its return is prevented." —Vide a Review of Dr. Caldwell's Essay, in the *Edinburgh Phrenological Journal,* vol. viii.

by painful and unmitigated suffering; and being surrounded by many temptations of hunger and comparative nudity, they are often stimulated to commit offences against the laws, from a mere sense of self-preservation. And if they avoid such acts, they are still liable to be the victims of impulses, and sacrifice, for present gratification, purity and self-respect. Girls are more to be pitied than boys, as, without knowledge or means, they soon fall victims to prurient appetites.

Although our subject rendered these contrasts a necessary result of our investigation of consequences, yet we cannot help concluding that the offspring of the inveterately intemperate, whether wealthy or needy, cannot and do not escape from some moral blight. The sons of poverty may present the coarser aspects, but the children of the better classes do not escape from the contaminating influence; they may be restrained from glaring manifestations, but they cannot escape altogether the "sins of their fathers."

Let us then use every effort to promulgate sound views on the effects of intemperance, so as to counteract and neutralize the moral poison which, like a blight, is gradually destroying the bodies and minds of thousands; and one of the most effectual modes would be, to treat drunkenness as a special form of insanity.

It would be merciful to do so when persons are incapable to resist temptation, and who, when once inebriated, commit the greatest outrages against all decorum, and violate all the laws of morality. The following example was published in a recent paper:*—

"On Wednesday, Margaret Broughton was brought before Mr. Jardine, charged with being a prostitute, and behaving disorderly in Holborn, and assaulting the police. Constable Madden, of the E division, found the prisoner, at a late hour last night, quarrelling with a cabman in Holborn. She was shouting and making a great noise. He remonstrated with her, and requested her to go away; but she only made the more noise, and abused him with the foulest language. At length he was obliged to remove her to the station-house, when she struggled violently, and struck him. She was drunk.

"The prisoner is the widow of a gentleman who died about seven years back, leaving her an income of 300*l.* a-year. She almost immediately abandoned herself to drinking and low company, and has ever since lived a deplorably profligate life. She draws her money quarterly, and in an incredibly short time squanders it away amongst her disreputable associates. Then, when all is spent, she goes on the town to support herself till the next quarter becomes due. She frequently

* *Dispatch*, November 22, 1857.

gets to prison, and at such times makes great promises of reform. These promises are sometimes kept for a short time after she comes out of prison, but as soon as quarter-day comes round she is sure to fall in with her 'friends,' who, of course, are on the watch for her, and who take good care not to leave her whilst she has a shilling.

"The prisoner was sentenced to three months' imprisonment. As she was being removed, she was heard to declare, that as soon as her term of punishment expired, she would take a cab from the prison door to her lawyer's office, take the quarter's money, which would fall due in the meanwhile, and go away out of town, from her old haunts and temptations."

A glance at the statistics of crimes perpetrated under the influence of partial intoxication, would warrant some stringent means. The murders, rapes, suicides, savage assaults, robberies, and so forth, all stand as fearful evidence that intemperance only stimulates the animal impulses; and we cannot but think that the greatest sticklers for the "freedom of the subject" must be forced to acknowledge, *that it is better to prevent the commission of crimes by forcibly restraining men who have lost all self-control, and who, without such extraneous influence, must steep their souls in deeper guilt!*

Which, we ask, is most in accordance with the true function of an enlightened Government—to suffer the rank growth of immorality to spread so luxuriantly, and jeopardize life, property, and the very stability of the community; or to prevent these consequences, by forcibly restraining offenders from wilfully and deliberately destroying themselves, and inflicting vast evils on others not implicated in their conduct?

There would not be any stretch of power in these conservative means. Few can plead ignorance as to the certain consequences of intemperance—consequences which, though modified by the different organizations of individuals, are never productive of noble and exalted actions. If, therefore, persons make the declaration that they cannot avoid an intemperate indulgence in alcoholic drinks, with all their fostering tendency to crimes, we repeat, they should be placed under restraint, and treated as patients, so that means might be applied to effect their recovery from the morbid influence of a degrading and most pernicious vice.

This treatment would be justifiable on other grounds, as it would save the children of drunken parents, who are almost always driven to the commission of crime, and thus aid in further polluting the moral atmosphere. What otherwise could be expected, when all the example set them is idleness, dissipation, and the worst form of selfishness?

If the present do-nothing state of things continue, we must be prepared for greater physical deterioration and moral degradation among the masses. For it follows, that if a vast many have inherited the fatal tendency, without some positive remedial treatment they will become more deadly than the fabled Upas tree!

The humblest mechanic may learn enough of chemistry and physiology, in the institutions devoted to his improvement and relaxation, to render him sufficiently well-informed of the destructive consequences of intemperance. He sees written on the brows of the inebriate the branded marks of either criminal depravity or pauper degradation.

If a comparatively educated man is more amenable than one left in extreme ignorance, how much greater, then, is the criminality of those who are early indoctrinated in the sacred truths of religion and a knowledge of moral responsibility, if they prostrate themselves before the idol of intemperance, and lose all the conservative influences of their early culture — if they have become indifferent to the priceless gifts of a conscience void of offence! Yet such persons must feel occasional pangs when beholding the debilitated minds and diseased bodies of their unfortunate offspring. And when the fatal truth is forced on their consciousness, that they are the authors of these sad calamities, can we wonder that, in a moment of moral nausea, they sever "the silver cord," and thus finish their vicious career by the presumptuous act of self-murder?

Lastly. We may confirm the actual propriety of treating intemperance as a form of insanity, by the fact that we have heard a vast many criminals, confined in different prisons, declare "that had they never tasted the 'fatal poison,' they might have remained industrious and respectable members of society."

Then what apology can be made for the *national* conscience, that there is not made some vigorous attempt to prevent the evil consequences, by removing the landmarks of temptation to inebriety, by shutting up "The Palaces," with their glaring lights, which necessarily attract the thoughtless, like flies, to their certain destruction?

We know that some efforts have been made; but they have either been too feeble, or else the clamour of "vested rights" have so bewildered our legislators, that they have not recognised the many crimes which are concocted in such haunts of vice!

Then, again, shallow political economists have boldly asserted that the revenue arising from the sale of intoxicating drinks cannot be dispensed with, even admitting that there are many evil consequences! They forget in this calculation the cost of criminals, and the tax which intemperance inflicts by the pauperism it occasions. The expenditure for these joint consequences is indeed enormous. And if, on striking a balance between the receipts, should it even be proved that the fiscal advantages of intemperance still leaves a surplus (which we deny)—then, even then we should pronounce any revenue obtained through such unhallowed means rather as a source of disgrace to an enlightened community than as a subject of congratulation!

We venture to add, that if we remain supine, and do not make any efforts commensurate to prevent the horrid injuries inflicted by intemperance, we must build more infirmaries, more dispensaries, more workhouses, more prisons, and more madhouses; and that for the very purpose of filling them with self-immolated victims, who blindly follow their insatiable appetites, unmindful of the injury they inflict on themselves, their unfortunate offspring, and on the community.

We are told of enthusiastic and besotted idolators worshipping the idol Juggernaut by throwing themselves under the wheels of its car, to which is attached scythes, which mow down these senseless beings by thousands, and we shudder at their barbaric ignorance; but is it not still more surprising that we, who boast of our religion, know that thousands are annually destroyed by alcohol, and we regard it as a matter of course, without making a vigorous effort to prevent such wholesale destruction?

The latter insensate devotees cannot plead ignorance, as the first-mentioned might do, for they know they *must* be sacrificed; and yet they go madly on, paying homage to this enemy of human happiness. They sin with their eyes open, and with a knowledge of the consequences of *their* idolatry. If they will not or cannot turn away from this fatal infatuation, let us forcibly prevent their continuing in a career which must lead to sin, disease, and a moral death; let us place them under restraint, and cure them of their insane affection.

Let us not be lukewarm on the subject, for the picture of intemperance we have sketched is neither embellished nor exaggerated. The scenes of crime, misery, disease, and destitution, in every locality, are the vouchers of our accurate delineation of its consequences. Then let us lay our hands on our hearts, and before God and our consciences declare that we will use every means to stop the onward tide of crime which flows from the polluted sources of intemperance; if we do not do so, we must stand charged with moral complicity. It is not any use merely to lament the evils which flow from it—we must put our shoulders to the wheel, and then pray for help; and to do this, we must show our deep abhorrence of its bitter fruits, not only by precepts, but by our own example; and then, indeed, we may regard success as certain.

In conclusion, we may remark that our recommendation of restraining the drunkard from continuing in his senseless and destructive career, is not a novel suggestion; an article on this subject appeared in one of the early numbers of the "Psychological Journal." In this article it was intimated that some place of refuge, or temporary asylum, differing from the usual lunatic asylums, would prove of real advantage to the community, and a blessing to those whose habits of intemperance would render it necessary to seclude them, as by such an arrangement they might be put under a course of judicious remedial treatment, and might soon recover their perfect normality of mind.

If, therefore, this suggestion was carried out into practice, the benefit would be incalculable, whilst religion and morality then would dispense their hallowed peace-giving tendency, and the truly humane would then be spared the many painful spectacles which originate solely through excessive intemperance.

Among the immediate advantages which would result, might be calculated a great diminution of crime; and amongst the working classes they would, with habits of sobriety, manifest greater prudence and forethought; and thus, with a sense of self-respect, would be found a great decrease of pauperism and its consequent degradation. And those amongst the wealthy, when they are cured of an insatiable habit, they would be more likely to attend to works of utility, and may thus become benefactors of their less fortunate fellow-subjects, instead of perverting their morals by their previous most debasing example. Thus we may affirm, that the good which would result from forcibly counteracting the inordinate craving tendency of chronic intemperance would be greater than the most sanguine spirits, in the full activity of their benevolence, could ever have anticipated.

And, therefore, as it is our firm conviction that mere precepts, however good, will not correct the errors and crimes resulting from drunkenness, then, in the name of religion, morality, patriotism, and humanity, there is demanded some forcible and effective means to render certain this essential and all-important reform in the habits of a vast proportion of the community.

